# Differential Targeting of Hsp70 Heat Shock Proteins HSPA6 and HSPA1A with Components of a Protein Disaggregation/Refolding Machine in Differentiated Human Neuronal Cells following Thermal Stress

**DOI:** 10.3389/fnins.2017.00227

**Published:** 2017-04-24

**Authors:** Catherine A. S. Deane, Ian R. Brown

**Affiliations:** Department of Biological Sciences, Centre for the Neurobiology of Stress, University of Toronto ScarboroughToronto, ON, Canada

**Keywords:** HSPA1A (Hsp70-1), HSPA6 (Hsp70B'), HSPA8 (Hsc70), DNAJ (Hsp40), HSPH1 (Hsp105α), human neuronal SH-SY5Y cells

## Abstract

Heat shock proteins (Hsps) co-operate in multi-protein machines that counter protein misfolding and aggregation and involve DNAJ (Hsp40), HSPA (Hsp70), and HSPH (Hsp105α). The HSPA family is a multigene family composed of inducible and constitutively expressed members. Inducible HSPA6 (Hsp70B') is found in the human genome but not in the genomes of mouse and rat. To advance knowledge of this little studied HSPA member, the targeting of HSPA6 to stress-sensitive neuronal sites with components of a disaggregation/refolding machine was investigated following thermal stress. HSPA6 targeted the periphery of nuclear speckles (perispeckles) that have been characterized as sites of transcription. However, HSPA6 did not co-localize at perispeckles with DNAJB1 (Hsp40-1) or HSPH1 (Hsp105α). At 3 h after heat shock, HSPA6 co-localized with these members of the disaggregation/refolding machine at the granular component (GC) of the nucleolus. Inducible HSPA1A (Hsp70-1) and constitutively expressed HSPA8 (Hsc70) co-localized at nuclear speckles with components of the machine immediately after heat shock, and at the GC layer of the nucleolus at 1 h with DNAJA1 and BAG-1. These results suggest that HSPA6 exhibits targeting features that are not apparent for HSPA1A and HSPA8.

## Introduction

Heat shock proteins (Hsps) are highly conserved proteins that play roles in cellular repair and protective mechanisms (Muchowski and Wacker, 2005; Asea and Brown, 2008; Paul and Mahanta, 2014). They co-operate in multi-protein machines to counteract protein misfolding and aggregation that are characteristic of neurodegenerative diseases (Muchowski and Wacker, 2005; Rampelt et al., 2012; Duncan et al., 2015; Nillegoda and Bukau, 2015; Nillegoda et al., 2015; Smith et al., 2015; Goloubinoff, 2017; Jackrel and Shorter, 2017). Misfolded proteins are detected by DNAJs (Hsp40s) and refolded into biologically active states by members of the HSPA (Hsp70) family (Rampelt et al., 2012; Mattoo and Goloubinoff, 2014; Clerico et al., 2015; Nillegoda and Bukau, 2015; Nillegoda et al., 2015). While Hsp70 and Hsp40 co-operate to prevent aggregation of misfolded proteins, they cannot dissociate protein aggregates that accumulate during neurodegenerative diseases (Rampelt et al., 2012; Gao et al., 2015; Nillegoda and Bukau, 2015; Nillegoda et al., 2015).

Yeast cells express a well-characterized “disaggregase” (Hsp104) that is able to solubilize aggregated proteins, homologs of which are lacking in mammalian cells (Glover and Lindquist, 1998; Weibezahn et al., 2005; Bösl et al., 2006; Nillegoda and Bukau, 2015). Studies have shown that HSPH1 (Hsp105α), a member of the mammalian Hsp110 family, acts co-operatively with Hsp70/Hsp40 as a “disaggregase” to dissociate aggregated proteins (Rampelt et al., 2012; Nillegoda and Bukau, 2015; Nillegoda et al., 2015). It has been reported that the mammalian disaggregation/refolding machine dissociates amyloid fibrils of α-synuclein that are associated with Parkinson's disease (Gao et al., 2015). Misfolded protein aggregates accumulate during the course of neurodegenerative diseases and upregulation of Hsps is being investigated as a potential protective strategy (Asea and Brown, 2008; Genc and Özdinler, 2014; Kalmar et al., 2014; Paul and Mahanta, 2014; Deane and Brown, 2016; Kampinga and Bergink, 2016).

The HSPA family is a multigene family composed of inducible and constitutively expressed members (Morimoto, 2008). HSPA6 (Hsp70B') is an inducible member that has received little attention compared to the more widely studied HSPA1A (Hsp70-1). HSPA6 has been investigated in cultured human neuronal cells (Chow and Brown, 2007; Chow et al., 2010; Khalouei et al., 2014a,b; Deane and Brown, 2016, 2017; Shorbagi and Brown, 2016; Becirovic and Brown, 2017), and in human cancer cell lines (Noonan et al., 2007, 2008). Interestingly, the HSPA6 gene is found in the human genome but not in mouse and rat, hence it is absent in current animal models of neurodegenerative diseases (Noonan et al., 2007; Deane and Brown, 2016, 2017).

To advance knowledge of HSPA6, we investigated whether it is targeted to stress-sensitive neuronal sites with components of a protein disaggregation/refolding machine in human neuronal SH-SY5Y cells that have been previously used as a model in studies of neurodegenerative diseases (Grynspan et al., 1997; Imamura et al., 2006; Ross and Spengler, 2007; Cheung et al., 2008; Plowey et al., 2008; Krishna et al., 2014). Neurodegenerative diseases affect differentiated neurons of the adult central nervous system, hence SH-SY5Y cells were differentiated in the present study by treatment with retinoic acid which results in inhibition of cell division and stimulates the development of neuronal processes (Jacobs et al., 2006; Ross and Spengler, 2007; Cheung et al., 2008). Retinoic acid is required for adult neurogenesis in the rat brain (Jacobs et al., 2006; Bonnet et al., 2008) and for maintenance of the differentiated state of dopaminergic neurons in the nigrostriatal pathway (Maden, 2007). The present studies suggest that HSPA6 exhibits features in its targeting that are not observed for the widely studied HSPA1A.

## Materials and methods

### Cell culture and differentiation

Human neuronal SH-SY5Y cells (American Type Culture Collection, Manassas, VA, USA) were cultured in Dulbecco's modified Eagle's medium (DMEM; Wisent, QC, Canada) with 10% fetal bovine serum (FBS; Wisent) at 37°C in a humidified 5% CO_2_ atmosphere. Cells were plated at 4.5 × 10^4^ cells per cm^2^ on coverslips placed inside 6-well plates and allowed to settle onto the growth surface and adhere for 24 h. Neuronal differentiation was induced by treatment with 10 μM all-*trans*-retinoic acid (R2625; Sigma Aldrich, St. Louis, MO, USA) for 72 h under serum free conditions.

### Induction of hsps and heat shock treatment

Following 72 h of differentiation, media containing all-*trans*-retinoic acid was removed and replaced with fresh serum free DMEM with 0.3 μM celastrol plus 50 μM arimoclomol to induce Hsps (Deane and Brown, 2016, 2017). Celastrol (70950; Cayman Chemical, Ann Arbor, MI, USA) dissolved in DMSO was added directly to the media. Arimoclomol (gift from Professor Michael Cheetham, Institute of Ophthalmology, University College London, UK) was prepared fresh for each experiment by dissolving in serum free DMEM and filtering. DMSO was used as a vehicle control for celastrol. Following 12 h incubation to facilitate Hsp induction, cells were fixed for immunofluorescence (no HS) or exposed to heat shock (HS). For heat shock, cells were immersed in a water bath calibrated at 43°C (± 0.2°C) for 20 min. Cells were then fixed for immunofluorescence (20 min) or returned to 37°C until being fixed at a later time during recovery (1 or 3 h). The commencement of heat shock represents *t* = 0.

### Immunofluorescence

Cells were fixed with 4% paraformaldehye in phosphate buffered saline (pH 7.4) for 30 min, permeabilized with 0.1% triton X-100 and 100 mM glycine for 30 min, and then blocked with 5% FBS for 1 h before being incubated with primary antibodies overnight in 1% FBS. HSPA1A (SPA-810), HSPA6 (SPA-754), HSPA8 (SPA-815), DNAJB1 (SPA-400), and HSPB1 (SPA-803) antibodies were obtained from Enzo Life Sciences (Farmingdale, NY, USA). DNAJA1 [clone KA2A5.6] (ab3089), HSPH1 (ab109624), BAG-1 (ab7976), SC35 (ab11826), nucleophosmin (ab37659), and RNA polymerase II CTD repeat YSPTSPS (phospho S5) (ab5131) primary antibodies were purchased from Abcam (Toronto, ON, CA). Primary antibody for the nuclear speckle marker SON (HPA023535) was obtained from Sigma Aldrich. Cells were washed and incubated with Alexafluor® Donkey secondary antibodies (Molecular Probes, Life Technologies, Burlington, ON, CA) and then counterstained with 300 nM DAPI (Invitrogen, Life Technologies). Fluorescence images were acquired using a Quorum Wave FX-X1 spinning disk confocal microscope (Quorum Technologies, Guelph, ON, CA) outfitted with a high resolution Humamatsu Orca R2 camera (Humamatsu Photonics, Japan) and a Plan-APO 63x/1.4NA oil objective. Excitation lasers: 405, 491, 561, and 644 nm. Emission filters (nm/bandpass): 460/50, 525/50, and 593/40.

### Image processing and analysis

Image processing utilized Volocity 3D image analysis software (PerkinElmer, Waltham, MA, USA). ImageJ software (http://imagej.nih.gov/ij/) was employed for co-localization analysis using TIFF images exported from Volocity. Background subtracted images were used to generate intensity profile plots representing the fluorescence signal intensities for the indicated channels in a defined linear region using the RGB (red-green-blue) Profiler plugin. Images representative of 3 individual experiments are shown in which 25 cells were analyzed in coverslips harvested from each well of 6-well culture plates.

## Results

### Differential targeting of HSPA6 and HSPA1A in human neuronal cells following thermal stress

**Figure 1:**
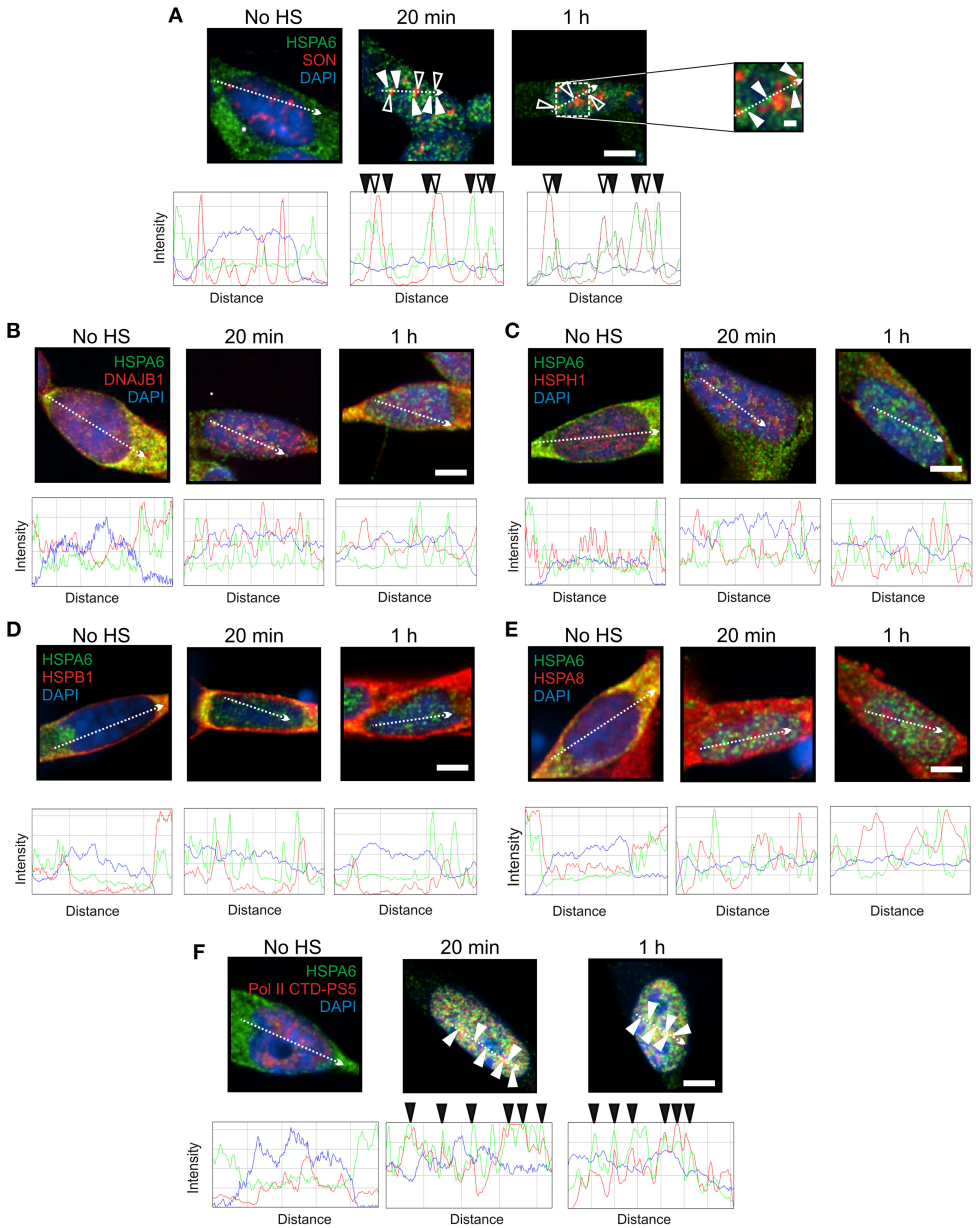
**HSPA6 was targeted to the periphery of nuclear speckles (perispeckles) following heat shock in differentiated human neuronal cells. (A)** Prior to heat shock, HSPA6 (green) was distributed in the neuronal cytoplasm. After heat shock, HSPA6 localized to foci at the periphery of nuclear speckles (closed arrowheads) identified by the marker protein SON (red, open arrowheads). DAPI (blue) was used to identify neuronal nuclei. ImageJ line scans demonstrated that HSPA6 fluorescent peaks were offset from SON peaks. ImageJ line scans confirm that **(B)** DNAJB1, **(C)** HSPH1, **(D)** HSPB1, and **(E)** HSPA8 did not co-localize with HSPA6. **(F)** HSPA6-positive foci co-localized with the perispeckle marker RNA polymerase II (closed arrowheads). Scale bar represents 5 μm **(A–F)**. Inset scale bar in **(A)** represents 0.5 μm.

**Figure 2:**
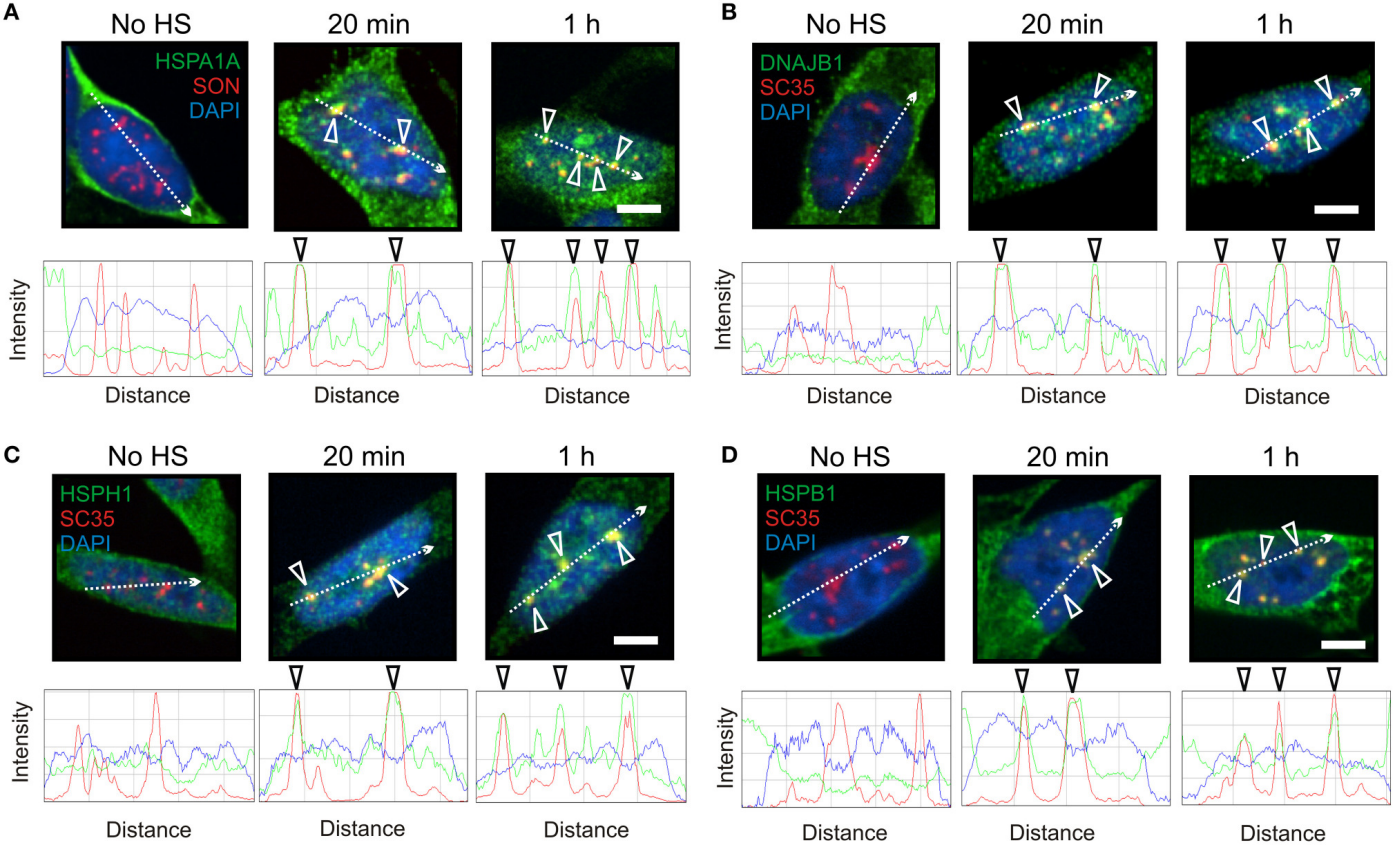
**HSPA1A co-localized at nuclear speckles with components of a mammalian protein disaggregation/refolding machine after thermal stress. (A)** Prior to heat shock, HSPA1A (green) was distributed in the neuronal cytoplasm. After heat shock, HSPA1A co-localized with the nuclear speckle marker protein SON (red, open arrowheads) at 20 min and 1 h, confirmed by ImageJ line scans. DAPI (blue) was used to identify neuronal nuclei. Components of a mammalian protein disaggregation/refolding machine including **(B)** DNAJB1, **(C)** HSPH1, and **(D)** HSPB1, also targeted nuclear speckles (open arrowheads) after heat shock at 20 min and 1 h, as determined by co-localization with nuclear speckle markers SON (HSPA1A) and SC35 (HSPH1, DNAJB1, and HSPB1). Scale bar represents 5 μm.

To induce Hsps, including HSPA6 (Hsp70B') and HSPA1A (Hsp70-1), differentiated human neuronal SH-SY5Y cells were treated with celastrol and arimoclomol as previously described (Deane and Brown, 2016). HSPA6 and HSPA1A were distributed in the neuronal cytoplasm prior to heat shock (Figures 1A, 2A, No HS panels). At 20 min and 1 h after heat shock, HSPA6 localized to perispeckles (Figure 1A, closed arrowheads) around the periphera of nuclear speckles (open arrowheads) which were identified with the SON marker protein (Sharma et al., 2010; Sytnikova et al., 2011; Khalouei et al., 2014b). As shown in Figures 1B–E, components of the mammalian disaggregation/refolding machine, namely DNAJB1 (Hsp40-1), and the “disaggregase” HSPH1 (Hsp105α), and also HSPB1 (Hsp27) and HSPA8 (Hsc70), did not co-localize after heat shock with the HSPA6-positive perispeckles, confirmed by ImageJ line scans located below the immunocytochemistry panels. HSPA6 co-localized with the perispeckle marker RNA polymerase II (Figure 1F) that is associated with transcription sites (Ghamari et al., 2012).

As shown in Figure 2, components of the disaggregation/refolding machine were targeted with HSPA1A to nuclear speckles, as determined by co-localization with nuclear speckle markers SON and SC35 (Figure 2, open arrowheads). The SC35 and SON antibodies used in the present study have been shown to co-localize at nuclear speckles that are enriched in RNA splicing factors in differentiated human neuronal SH-SY5Y cells (Lamond and Spector, 2003; Spector and Lamond, 2011; Khalouei et al., 2014b). ImageJ line scans confirmed the co-localization of HSPA1A at nuclear speckles with DNAJB1 (Figure 2B), HSPH1 (Figure 2C), and HSPB1 (Figure 2D). Hence, HSPA1A was targeted with components of a protein disaggregation/refolding machine to nuclear speckles after heat shock (Figure 2), while HSPA6 localized to perispeckles, where signal for these machine components was not detected (Figure 1).

### Association of HSPA1A and HSPA6 with the nucleolus in neuronal cells

**Figure 3:**
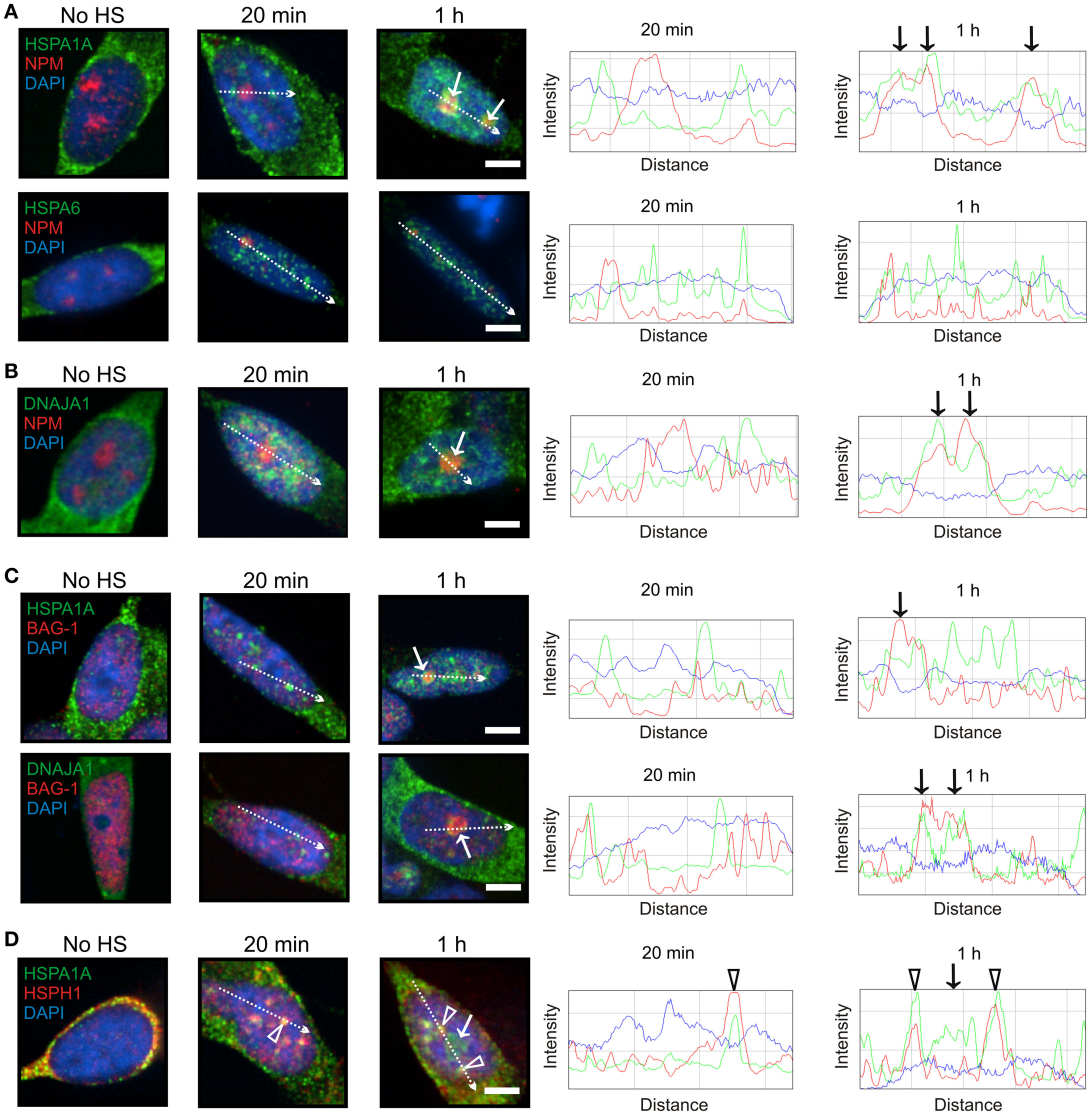
**HSPA1A, but not HSPA6, was targeted to the GC layer of the nucleolus at 1 h following heat shock. (A)** At 1 h, HSPA1A (green, upper panel), but not HSPA6 (green, lower panel) co-localized with nucleophosmin (NPM) (red, arrow), a marker of the GC layer of the nucleolus. This localization was not observed at 20 min, confirmed by ImageJ line scans shown on the right. **(B)** DNAJA1 also co-localized with nucleophosmin at 1 h (arrows). **(C)** BAG-1 co-localized at 1 h with HSPA1A and DNAJA1 (arrows) that were shown to localize to the nucleolus in **(A,B)**, however the “disaggregase” HSPH1 did not (**D**, arrow). The open arrowheads represent HSPA1A and HSPH1 targeting to nuclear speckles, previously shown in Figures 2A,C. Scale bar represents 5 μm.

At 1 h after heat shock, HSPA1A, but not HSPA6, was targeted to the granular component (GC) of the nucleolus (Figure 3A, arrows), identified by nucleophosmin (NPM) marker protein (Hernandez-Verdun et al., 2010), which is the site of ribosomal RNA processing and ribosomal subunit assembly (Thiry and Lafontaine, 2005; Raska et al., 2006; Hernandez-Verdun et al., 2010). DNAJA1 also co-localized with nucleophosmin at the GC layer of the nucleolus (Figure 3B, arrows). As shown in Figure 3C (arrows), BAG-1 co-localized with HSPA1A and DNAJA1 which were targeted to the nucleolus at 1 h (Figures 3A,B, arrows), whereas the “disaggregase” HSPH1 did not (Figure 3D, arrow). The open arrowheads in Figure 3D represent the targeting of HSPA1A and HSPH1 to nuclear speckles, previously mentioned in Figures 2A,C.

**Figure 4:**
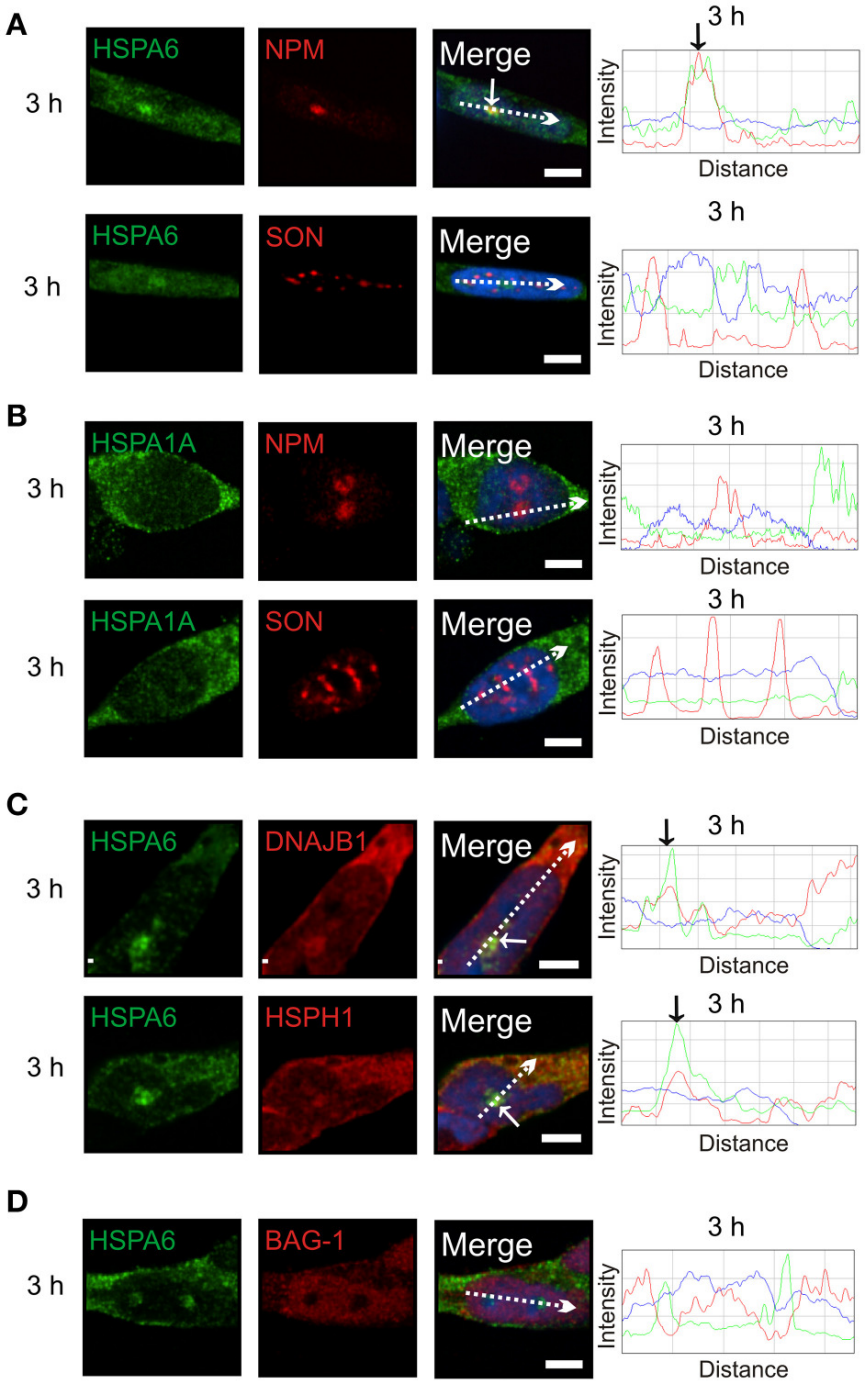
**At 3 h after thermal stress, HSPA6 co-localized with nucleophosmin, a marker of the granular component (GC) of the nucleolus. (A)** HSPA6 co-localized at the 3 h time point with a marker of the GC layer of the nucleolus, nucleophosmin (arrow, upper panel), but not with the nuclear speckle marker SON (open arrowheads, lower panel). **(B)** HSPA1A did not co-localize with nucleophosmin (upper panel) or SON (lower panel) at 3 h. **(C)** Components of a mammalian disaggregation/refolding machine, including DNAJB1 and HSPH1, were also targeted to the GC layer of the nucleolus at 3 h (arrows), however **(D)** BAG-1 was not. DAPI (blue in merged panels and ImageJ line scans) was used to identify neuronal nuclei. Scale bar represents 5 μm.

Subsequently at 3 h after heat shock, HSPA6 (Figure 4A, arrow), but not HSPA1A (Figure 4B), co-localized at the GC layer of the nucleolus with components of a protein disaggregation/refolding machine, namely DNAJB1 and the “disaggregase” HSPH1 (Figure 4C, arrows), but, interestingly, not BAG-1 (Figure 4D). These results suggest differential targeting of HSPA6 and HSPA1A to nucleolar structures following thermal stress.

### Constitutively expressed HSPA8 exhibited similar heat shock-induced targeting as HSPA1A, however HSPA6 did not

HSPA8 (Hsc70) is a constitutively expressed member of the HSPA family that is expressed at high levels in neurons compared to other cell types and has been proposed to provide pre-protection from neuronal stress (Manzerra et al., 1993, 1997; Chen and Brown, 2007a,b). Inducible HSPA members, particularly HSPA1A, have been more widely investigated in studies of protein misfolding and aggregation resulting from cellular stress. However, it has been recognized that constitutive Hsps, including HSPA8, also have stress-related functions (Manzerra et al., 1993; Vos et al., 2008; Stricher et al., 2013).

**Figure 5:**
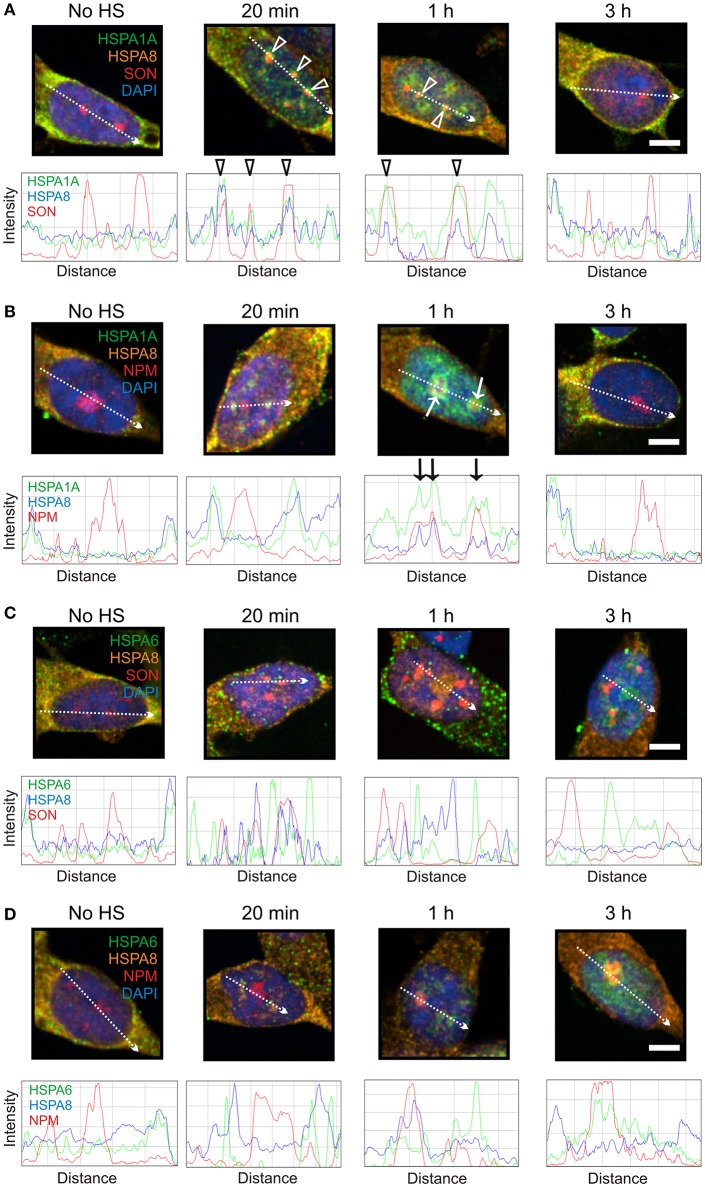
**Constitutively expressed HSPA8 exhibited similar heat shock-induced targeting as HSPA1A, however HSPA6 did not. (A)** HSPA8 targeted SON-positive nuclear speckles at 20 min and 1 h after heat shock. **(B)** HSPA8 also targeted the GC layer of the nucleolus (identified by the marker protein nucleophosmin) at 1 h and co-localized with HSPA1A. **(C)** HSPA8 did not co-localize with HSPA6 at 20 min and 1 h at perispeckles or **(D)** at the GC layer of the nucleolus at 3 h. DAPI (blue) was used to identify neuronal nuclei. Scale bar represents 5 μm.

As shown in Figure 5, HSPA8 localized to SON-positive nuclear speckles at 20 min and 1 h after heat shock (Figure 5A, open arrowheads), and to the nucleophosmin-positive GC layer of the nucleolus at 1 h (Figure 5B, arrows), before returning to the cytoplasm at 3 h. This pattern of heat-induced targeting to neuronal sites was similar to that of HSPA1A (Figures 5A,B), but not HSPA6 (Figures 5C,D). These results indicate that HSPA8 exhibits similar targeting after thermal stress as inducible HSPA1A. In contrast, HSPA6 exhibits features that are not observed for HSPA1A and HSPA8.

## Discussion

HSPA6 (Hsp70B') and HSPA1A (Hsp70-1) are inducible members of the HSPA (Hsp70) family (Chow and Brown, 2007; Noonan et al., 2007, 2008; Deane and Brown, 2016). We have previously shown that these proteins are not detectable in differentiated human neuronal SH-SY5Y cells but are induced by low dose co-application of celastrol and arimoclomol at concentrations that do not affect cell viability (Deane and Brown, 2016). Dividing human tissue culture cell lines, such as unstressed HeLa cells, express high basal levels of HSPA1A (Finka and Goloubinoff, 2013). However, this is not observed in unstressed, differentiated human neuronal SH-SY5Y cells which are non-dividing (Deane and Brown, 2016). The HSPA6 gene is present in the human genome, and in the marmoset monkey (NCBI gene ID: 100411854), camel (Elrobh et al., 2011) and goat (Banerjee et al., 2014) but is not found in the genomes of mouse and rat (Parsian et al., 2000), hence it is absent in current animal models of neurodegenerative diseases (Chow and Brown, 2007; Deane and Brown, 2016, 2017).

In order to advance knowledge of the little studied HSPA6, the present study investigated whether it is targeted to stress-sensitive neuronal sites with components of a mammalian disaggregation/refolding machine. Following thermal stress, HSPA1A, but not HSPA6, rapidly co-localized to nuclear speckles with DNAJB1 and HSPH1 components of a disaggregation/refolding machine. Nuclear speckles are rich in RNA splicing factors and splicing is inhibited by heat shock (Lamond and Spector, 2003; Spector and Lamond, 2011). In contrast, HSPA6, but not HSPA1A, was rapidly targeted by heat shock to perispeckles located at the periphera of nuclear speckles that are rich in RNA polymerase II and poly(A+)-containing RNA (Bregman et al., 1995; Mortillaro et al., 1996; Hall et al., 2006; Khalouei et al., 2014b) and have been characterized as “transcription factories” (Brown et al., 2008; Rieder et al., 2012, 2014). Interestingly, components of the disaggregation/refolding machine, namely DNAJB1, and the “disaggregase” HSPH1 (Hsp105α), did not co-localize with HSPA6 at perispeckles. This suggests a role for HSPA6 at perispeckles that does not require the elements of the disaggregation/refolding machine. It has been reported that HSPA6 is capable of refolding heat-denatured p53 in the absence of DNAJ proteins (Hageman et al., 2011). Small heat shock proteins (sHsps) have been reported to enhance recovery from heat-induced nuclear protein aggregation (Kampinga et al., 1994; Stege et al., 1995) likely by maintaining denatured proteins in a folding competent state (Ehrnsperger et al., 1997; Lee et al., 1997; Deunnwald et al., 2012; Rampelt et al., 2012). The present results indicate that HSPB1 (Hsp27) co-localized at nuclear speckles after heat shock with disaggregation/refolding machine components including HSPA1A, DNAJB1, and HSPH1.

Later in the time course after heat shock, HSPA6 and HSPA1A are differentially targeted to the GC layer of the nucleolus which is involved in ribosomal RNA processing and ribosomal subunit assembly (Thiry and Lafontaine, 2005; Raska et al., 2006; Hernandez-Verdun et al., 2010). At the 1 h recovery time point, HSPA1A, but not HSPA6, co-localized at the GC layer of the nucleolus with DNAJA1 and BAG-1, but not with HSPH1 (Hsp105α). BAG-1 targets Hsp70 substrates to the proteasome to facilitate their degradation (Bracher and Verghese, 2015a,b) and does not promote the dissociation of protein aggregates in the presence of other members of the disaggregation/refolding machine (Rampelt et al., 2012). This suggests a possible role for HSPA1A in BAG-1-directed targeting of heat damaged nucleolar proteins to the proteasome for degradation, which is not observed for HSPA6. Subsequently at the 3 h recovery time point, HSPA6, but not HSPA1A, is targeted to the GC layer of the nucleolus with components of the disaggregation/refolding machine comprised of DNAJB1, and the ‘disaggregase’ HSPH1.

HSPA8 (Hsc70) is a constitutively expressed member of the HSPA (Hsp70) family that is present at high levels in neurons in the mammalian brain (Manzerra et al., 1997). It has been proposed that HSPA8 may pre-protect neurons from stress (Chen and Brown, 2007a,b). The present results indicate that following thermal stress, constitutively expressed HSPA8 is targeted to nuclear speckles with components of the disaggregation/refolding machine. This suggests that neurons may have the capacity to rapidly form a protein disaggregation/refolding machine without the time lag needed to induce HSPA1A. Enhancing levels of HSPA8 could represent an additional strategy to combat protein misfolding and aggregation. The current studies reveal that HSPA8 exhibits targeting features that are similar to HSPA1A and different from HSPA6, that is, (i) co-localization at nuclear speckles with machine components and (ii) targeting to the GC layer of the nucleolus with BAG-1.

Therapies for neurodegenerative diseases that showed promise in current animal models have failed to translate effectively in human clinical trials suggesting deficiencies in these animal models (Nestler and Hyman, 2010; Lang, 2010; Dunkel et al., 2012; t Hart et al., 2012; Sheikh et al., 2013; McGonigle and Ruggeri, 2014; Sasaki, 2015). The present results suggest that elements of the cellular stress response, involving targeting of HSPA6 to perispeckles and later to the GC layer of the nucleolus at 3 h, that are present in differentiated human neuronal SH-SY5Y cells, are absent in current mouse and rat models of neurodegenerative diseases that lack the HSPA6 gene. Primate models are currently being developed using the common marmoset, an animal that possesses the HSPA6 gene (NCBI gene ID: 100411854) (Lang, 2010; t Hart et al., 2012; McGonigle and Ruggeri, 2014; Sasaki, 2015).

## Author contributions

CD and IB carried out the design of the work, data acquisition and data analysis. CD and IB also contributed to the writing of the manuscript, gave final approval of the version to be published, and agreement to be accountable for all aspects of the work.

## Funding

Supported by grants from NSERC to IB.

### Conflict of interest statement

The authors declare that the research was conducted in the absence of any commercial or financial relationships that could be construed as a potential conflict of interest.
